# Smart Hydrogel for the Treatment of Rheumatoid Arthritis

**DOI:** 10.3390/gels12030209

**Published:** 2026-03-04

**Authors:** Wenfeng Jiao, Xueya Wang, Hui Xu, Yang Fei, Yong Jin

**Affiliations:** School of Pharmacy, Anhui Medical University, Hefei 231200, China; 2545011119@stu.ahmu.edu.cn (W.J.); 2545011079@stu.ahmu.edu.cn (X.W.); 2445010846@stu.ahmu.edu.cn (H.X.); 2445011029@stu.ahmu.edu.cn (Y.F.)

**Keywords:** rheumatoid arthritis, smart hydrogel, responsive release mechanism, inflammatory pathway, immune microenvironment

## Abstract

Rheumatoid arthritis (RA) is a chronic autoimmune disease that imposes substantial physical, emotional, and socioeconomic burdens on patients. Conventional therapeutic approaches are often limited by systemic toxicity, inadequate joint targeting, and variable patient responses, highlighting the urgent need for advanced drug delivery systems. Smart hydrogels have emerged as a promising platform for RA treatment due to their unique three-dimensional hydrophilic networks, excellent biocompatibility, and tunable physicochemical properties. This review systematically summarizes the preparation strategies and design principles of smart hydrogels, with an emphasis on chemically and physically crosslinked networks as well as composite systems. It further outlines the major stimulus-responsive release mechanisms—including temperature, pH, reactive oxygen species (ROS), light, and enzyme triggers—that enable targeted and controlled drug delivery within the inflamed joint microenvironment. Among the various types discussed, temperature-responsive and multi-responsive hydrogels are most frequently investigated for their potential to achieve localized, on-demand therapy. Despite considerable preclinical progress, the clinical translation of smart hydrogels faces critical challenges, including insufficient long-term biocompatibility data, lack of standardized evaluation protocols, and difficulties in scalable manufacturing. This review aims to provide a conceptual framework for the rational design of smart hydrogels and to stimulate interdisciplinary efforts toward overcoming existing translational barriers in RA treatment.

## 1. Introduction

Rheumatoid arthritis (RA) is a chronic autoimmune inflammatory disorder that primarily affects joints and the surrounding soft tissues, frequently culminating in joint dysfunction, deformity, or irreversible damage [1]. Characterized by inflammation, bone erosion, and cartilage degradation, RA can cause persistent joint impairment [2]. It has genetic associations with multiple sclerosis, inflammatory bowel disease, and type 1 diabetes, suggesting shared immune mechanisms [3]. Globally, approximately 1% of the population is affected by RA [4]. Among patients with early-stage RA, roughly 11% develop interstitial lung disease (ILD), with advanced age and moderate-to-high disease activity being significant risk factors [5], which imposes considerable physical, psychological, and socioeconomic burdens on patients.

Currently, the clinical management of rheumatoid arthritis (RA) primarily relies on medication. Disease-modifying antirheumatic drugs (DMARDs) serve as the foundation of treatment, aiming to alleviate pain and suppress inflammation [6]. To relieve inflammation, nonsteroidal anti-inflammatory drugs (NSAIDs) are utilized to ease pain and reduce swelling [7,8]. Biologics, such as inflammatory cytokine inhibitors (ICIs), specifically target inflammatory pathways to prevent the excessive production of inflammatory factors [9]. Meanwhile, targeted therapies, like those modulating the ubiquitination pathway, promote cartilage repair and influence disease progression [10]. However, current treatments face significant obstacles. They frequently fail to effectively address the root causes of RA or alter its trajectory [11]. Prolonged systemic use, including the use of biologics, can lead to drug resistance, resulting in reduced efficacy or causing relapses [12]. Moreover, substantial inter-patient variability, such as comorbidities and differences in drug response, complicates treatment selection and affects treatment outcomes [13]. These challenges drive the pursuit of more efficient, safer, and controllable drug delivery systems.

Hydrogel-based drug delivery systems exhibit substantial potential in the treatment of arthritis, attributable to their distinctive physicochemical characteristics and biocompatibility [14]. The three-dimensional, porous, and hydrophilic network of hydrogels facilitates the encapsulation of a wide variety of therapeutic agents, encompassing small molecules, biologics, and nucleic acid drugs, protecting them from premature degradation [15]. The pathogenesis of rheumatoid arthritis encompasses multiple cell types, such as synovial fibroblasts and chondrocytes [16,17], along with complex networks of pro-inflammatory cytokines, such as TNF-α and IL-6, and signaling pathways, including NF-κB [18]. Traditional delivery methods often fall short in ensuring targeted drug delivery, spurring the search for advanced carriers. Smart hydrogels, which are embedded with environment-responsive molecules, can accomplish precise drug release at inflamed joints in response to external stimuli such as pH or temperature [19]. Moreover, their outstanding biocompatibility and degradability reduce systemic toxicity to the minimum and enhance local drug concentration [20]. The research on smart hydrogels represents a significant advancement in joint-targeted therapy, presenting promising clinical applications [21].

## 2. Pathological Progression and Therapeutic Approaches of Rheumatoid Arthritis

### 2.1. Pathological Progression

#### 2.1.1. Molecular Mechanisms of Synovitis and Joint Destruction

Rheumatoid arthritis (RA) is a systemic autoimmune disorder marked by chronic synovial inflammation and progressive bone and cartilage damage. Synovitis in rheumatoid arthritis (RA) is marked by persistent inflammation, which is mainly driven by the abnormal proliferation of fibroblast-like synovial cells (FLS) and their secretion of inflammatory substances [22]. FLS actively participate in cartilage and bone degradation by releasing enzymes such as matrix metalloproteinases (MMPs) [23]. Senescence-associated secretory phenotype (SASP) refers to the extensive remodeling of the secretome during cellular senescence, characterized by the maintenance of high metabolic activity and secretion of numerous bioactive molecules by senescent cells after entering an irreversible cell cycle arrest state. These secretions include pro-inflammatory cytokines (e.g., IL-6, IL-8, IL-1), chemokines (e.g., CCL20), growth factors (e.g., VEGFA, TGF-β), proteases (e.g., MMP2, matrix metalloproteinases), bioactive lipids, extracellular vesicles, and various other soluble factors, exhibiting complex composition and high heterogeneity [24]. In the inflammatory synovial microenvironment, SASP continuously releases pro-inflammatory cytokines (including TNF-α and IL-6) in concert with matrix metalloproteinases (MMPs), leading to chondromatous matrix degradation and osteolysis [25]. Moreover, the interaction between macrophages and FLS further exacerbates joint destruction through the local release of inflammatory factors [26].

#### 2.1.2. Key Signal Pathways

**Figure 1:**
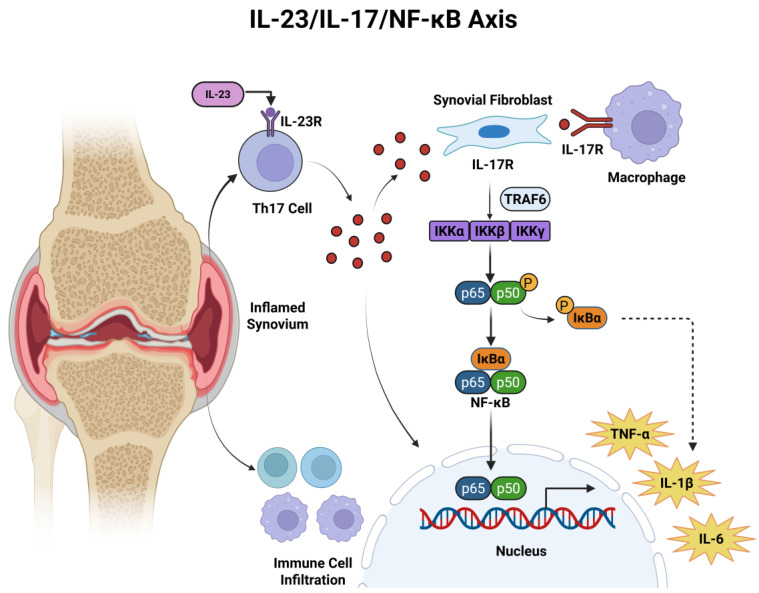
The IL-23/IL-17/NF-κB signaling pathway in the pathological progression of rheumatoid arthritis (RA), where IL-17R secretion stimulates abnormal proliferation of synovial fibroblasts and induces the release of pro-inflammatory factors (e.g., TNF-α, IL-6, IL-1β).

The IL-23/IL-17/NF-κB pathway is pivotal in rheumatoid arthritis (RA). As shown in Figure 1. IL-23 spurs Th17 cells to produce IL-17, which then activates NF-κB, triggering pro-inflammatory cytokine overproduction and joint inflammation [27]. The phosphorylation levels of p65 and IκBα are elevated in synovial tissue. Increased nuclear translocation of NF-κB drives the transcription of downstream inflammatory genes [28], exhibiting a multi-level and sustained activation that spans the entire process of inflammation initiation, synovial hyperplasia, tissue destruction, and deterioration of the immune microenvironment.

In rheumatoid arthritis (RA),the DNA sensor cGAS is abnormally activated in fibroblast-like synovial cells, driving extracellular trap formation and fostering tumor-like behavior in these cells [29]. This pathway is crucial for innate immune responses and inflammation. The TNF signaling pathway, a classic inflammatory route in RA, intertwines with other pathways like AGE-RAGE, HIF-1, and VEGF to form a complex inflammatory network [30]. TNF-α is pivotal in joint destruction within RA [31,32]. The JAK-STAT pathway, another key inflammatory mediator, influences immune cells and damaged tissues, with its abnormal activation triggering pro-inflammatory cytokine production [33]. Moreover, the interplay between BMP signaling and other pathways is vital for musculoskeletal disease development, such as RA [34], with interactions with the Wnt pathway being especially critical during bone erosion [35].

#### 2.1.3. Imbalance of RA Immune Microenvironment and Cytokine Networks

The rheumatoid arthritis microenvironment (RAM) is a mix of activated immune cells, like macrophages and T cells, and effector cells, including synovial and osteoclast cells [36]. A key imbalance in macrophage polarization—more pro-inflammatory M1 types versus fewer anti-inflammatory M2 types—disrupts the microenvironment, causing ongoing inflammation and joint damage [37]. A “gas imbalance phenomenon” may also exist in RAM, where the homeostasis of nitric oxide (NO) and reactive oxygen species (ROS, such as superoxide anion O_2_^−^) within cells is disrupted. This leads to excessive production of both substances and their harmful chemical reactions, thereby driving disease progression and treatment failure. The exacerbation of inflammation subsequently releases excess ROS, creating a vicious cycle of “excessive inflammation-gas imbalance-treatment resistance [38].” For instance, neighboring cells in the synovium shape the inflammatory setting through interactions, though the exact mechanisms are still unclear [39], complicating effective treatment.

### 2.2. Treatment Strategies for Rheumatoid Arthritis

#### 2.2.1. Current Status of RA Drug Therapy

Conventional drugs form the cornerstone of RA treatment, primarily including DMARDs, NSAIDs, and glucocorticoids. Methotrexate, a representative synthetic DMARD, is the first-line treatment for RA, used to delay joint destruction and disease progression [40]. NSAIDs are employed to alleviate pain and swelling and are commonly used as first-line therapy in clinical practice. Glucocorticoids such as prednisone exhibit potent anti-inflammatory effects and can rapidly control acute exacerbations [41]. Targeted therapies focus on specific molecular pathways to improve efficacy and reduce systemic side effects. These are often combined with biologic DMARDs targeting specific inflammatory factors (e.g., TNF-α, IL-6), providing more precise inflammation control in clinical settings [42]. For instance, tocilizumab effectively reduces inflammation and joint damage by inhibiting IL-6 receptors or directly blocking IL-6 [43]. Biologic DMARDs demonstrate favorable outcomes in patients who respond poorly to conventional DMARDs, filling the therapeutic gap for refractory RA.

#### 2.2.2. Limitations of Current RA Drug Therapy

RA, as a chronic inflammatory condition, requires high-dose, frequent, or long-term medication, which can lead to severe side effects (such as infections, hepatotoxicity, or cardiovascular events) and reduce patient compliance [44]. This is because all DMARDs—whether conventional drugs, biologics, or targeted therapies—exhibit immunologic nonselectivity. Systemic administration (e.g., oral or intravenous) often fails to achieve therapeutic concentrations at the joints due to uneven drug distribution and dose-limiting toxicity, resulting in limited efficacy and potential systemic side effects [45]. Consequently, researchers are exploring emerging therapeutic strategies to enable more precise drug targeting (e.g., modulating macrophage or synovial fibroblast behavior) and to alter drug loading and delivery methods to avoid the side effects associated with high-dose or long-term use. In addition to pharmacotherapy, current strategies include physical therapy, vaccine therapy, immunotherapy, and alternative therapies. Among these, hydrogels have talent showing itself for overcoming RA drug delivery challenges due to their responsive mechanisms. As an intra-articular local delivery system, smart hydrogels demonstrate differentiated advantages in targeting, controlled release, microenvironment modulation, and safety compared to injectable biologics, delayed-release reservoir formulations, or nanocarriers. For instance, in rat models, a single intra-articular injection of the hydrogel-based carrier achieved staged self-regulated release of methotrexate, with significantly superior anti-inflammatory effects compared to conventional biologic injections and delayed-release formulations. Additionally, smart hydrogels enhance drug retention time in the synovium through intra-articular delivery, reducing systemic exposure. In contrast, biologics (e.g., infliximab, tocilizumab) have shown an increased risk of pulmonary fungal infections in real-world data (ROR up to 26.02), with some drugs associated with fatal outcomes [46]. The hydrogel system, due to its localized action, can avoid such systemic adverse reactions. Based on these characteristics, hydrogels demonstrate potential as a complement and optimization to existing delivery systems in enhancing therapeutic efficacy, reducing systemic toxicity, and improving joint function, providing an innovative direction for the precision treatment of rheumatoid arthritis.

## 3. Preparation and Design Principle of Hydrogel

### 3.1. Chemical Crosslinking Method

Chemically cross-linked hydrogels create robust three-dimensional networks through chemical bonds, predominantly covalent. Polymer chains are permanently linked via chemical reactions, such as free radical polymerization, addition, or condensation, forming water-insoluble structures that absorb significant amounts of water or biological fluids [47]. Covalent cross-linking involves connecting molecular chains through covalent bonds, employing diverse designs like dynamic/static or light/enzyme-triggered reactions, and fostering interdisciplinary innovations to tailor hydrogel shapes and properties [48]. For example, collagen hydrogels use photoinitiator cross-linking to form covalent bonds between nanoparticles, enabling rapid gelation and injectability [49]. Step-growth chemistry utilizes multi-step reactions, such as azide-alkyne ring addition or thiol-ene reactions, ideal for high-biocompatibility applications as they avoid toxic catalysts and support multifunctional designs [50]. Specific reactions, like the Schiff base reaction in chitosan-gallic acid conjugates, spontaneously form gelatinous hydrogels under physiological pH, eliminating the need for extra crosslinking agents and making them suitable for 3D printing scaffolds [51,52]. Metal coordination, involving coordination bonds between metal ions and polymer ligands, enhances selectivity and mechanical strength, as seen in polyacrylic acid-acrylamide hydrogels [53]. These applications highlight the versatility and promise of chemically cross-linked hydrogels, especially in high-reliability, customized scenarios.

### 3.2. Physical Crosslinking Method

Physical crosslinking creates reversible network structures in hydrogels through self-healing secondary forces, granting them remarkable elasticity and adaptability. This method relies on physical interactions between polymers, such as electrostatic attractions between oppositely charged groups (e.g.,-NH_3_^+^ and -COOH), to form crosslinks [54]. During hydrogel synthesis, these electrostatic forces can shape network structures, influencing swelling and mechanical properties. Hydrogen bonds or physical entanglements between host and guest molecules in the material can also create crosslinking networks. When designing polysaccharide crosslinkers, incorporating physical entanglements boosts the hydrogel’s energy dissipation capacity [55]. Similarly, polyacrylamide [(C_3_H_5_NO)_n_] hydrogels enhance mechanical properties through hydrogen bonding and physical crosslinking with nanoscale clay [56]. Another physical crosslinking approach is dynamic, utilizing reversible non-covalent interactions to form dynamic and reversible crosslinking points [57]. For instance, glycerol (C_3_H_8_O_3_) or calcium ion-induced crosslinking can impart injectability and self-healing properties to hydrogels. Studies have demonstrated that sodium alginate [(C_6_H_7_O_6_Na)_n_] hydrogel fibers, ion-crosslinked with calcium chloride (CaCl_2_), exhibit high tensile strength (around 1.55 MPa) and fracture strain (approximately 161%) [58], showcasing the potential of physical crosslinking in creating robust yet adaptable hydrogel materials.

### 3.3. Preparation of Hydrogels by Composite Methods

Hydrogels, integrating various technologies, materials, and optimization strategies, often require a blend of chemical and physical cross-linking methods to enhance their performance, such as mechanical strength, biocompatibility, and functionalization, by harnessing the strengths of different components. This enhancement is typically achieved through two main strategies. The first strategy introduces organic compounds to speed up cross-linking. For instance, while polyvinyl alcohol (PVA) and hyaluronic acid (HA) composite hydrogels can be prepared via freeze–thaw cycles, this process is lengthy. Thus, small-molecule 3,4-dihydroxyphenylacetic acid (DHPA) is added to expedite cross-linking [59]. Similarly, in the field of polysaccharide-based composites, such as chitosan-starch blended systems, it is common to employ maleic acid as an effective chemical cross-linking agent. This approach is widely adopted due to its ability to enhance the structural integrity and functional properties of the composite materials. To achieve optimal performance, the precise ratio of maleic acid is carefully determined and fine-tuned through the application of response surface methodology, which allows for systematic optimization and ensures the best possible outcomes in terms of material strength and stability [60]. The second strategy incorporates nanoparticles, metal oxides, or other inorganic materials to boost functionality. For example, hydrogel composites with zinc oxide (ZnO) enhance mercury adsorption, while gelatin hydrogels with carbon nanofibers (CNFs), prepared through homogeneous dispersion, form conductive and printable 3D scaffolds [61,62]. Composite methods enable precise control of hydrogel mechanical properties by introducing diverse polymer chains or nanoparticles into the network, creating materials that are both strong and flexible to meet various application demands [63]. Additionally, these methods can impart unique stimulus-responsive properties, like reversible volume changes, to hydrogels [64], offering significant potential in biomedical and engineering fields, including drug delivery and smart sensors.

**Figure 2:**
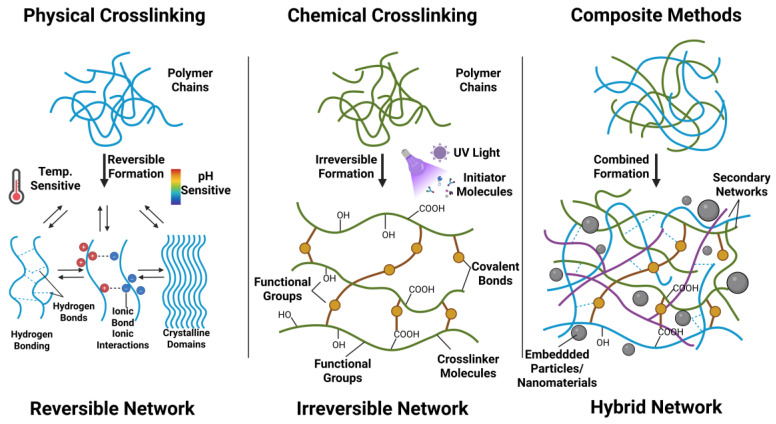
Hydrogel preparation process.

Figure 2 presents schematic diagrams of three preparation methods. Building on these established and innovative methods, a new generation of smart hydrogels that are highly responsive to diverse physiological environments has been successfully engineered and crafted. These advanced materials possess the remarkable capability to enable exceptionally precise and controlled drug release mechanisms, while simultaneously promoting effective tissue regeneration processes, particularly in the field of targeted drug delivery [65]. This significant technological advancement is spearheading the development of novel and more efficient strategies for treating a wide range of diseases, thereby opening up promising new avenues for therapeutic interventions and medical applications.

## 4. Release Mechanism of Smart Hydrogel

**Figure 3:**
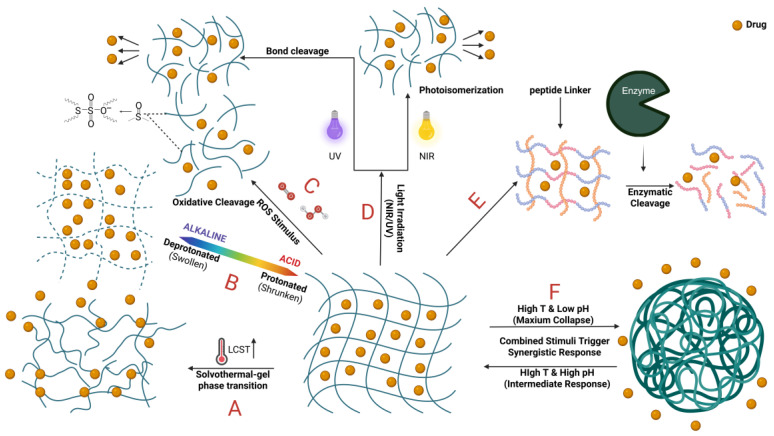
Mechanism of Smart Hydrogel Release. A. Temperature-responsive type. B. pH-responsive type. C. ROS-responsive type. D. Light-responsive type E. Enzyme-responsive type. F. Dual or multiple-responsive type.

Smart hydrogels are advanced materials that release therapeutic drugs in response to specific material–stimulus interactions. This process is dynamically regulated by either external or internal cues, which can include a wide range of physical, chemical, or biological factors [66]. These stimuli trigger changes in the hydrogel structure, enabling controlled and targeted drug delivery. Below, we outline some of the most common stimulus-response mechanisms utilized by these smart systems (Figure 3).

### 4.1. Temperature-Responsive Type

Temperature-responsive hydrogels, a type of thermoreactive molecularly imprinted hydrogel, control drug release via dynamic molecular binding sites that change conformation with temperature shifts. Below the transition temperature, drug leakage is minimized, above it, release accelerates [67]. This makes them ideal for temperature-triggered drug delivery. For example, poly (N-isopropylacrylamide) (NIPAAm) stays in a sol state at low temperatures (e.g., 25 °C), allowing easy injection and drug loading. At body temperature (e.g., 37 °C), it transitions to a gel, forming a localized drug reservoir for sustained release [68]. Besides morphological changes, these hydrogels can also adjust drug diffusion rates by expanding or contracting their polymer networks (e.g., ELP) near physiological temperatures. Sodium alginate–ELP hybrid hydrogels, for instance, regulate drug release through temperature-dependent swelling [69]. Combining different temperature-sensitive components, like NIPAAm and Pluronic 123, can shift the hydrogel’s low critical solubility transition (LCST), enabling hydrophilic drug loading at low temperatures and slow release at body temperature [70], broadening drug-loading options and release temperature ranges.

### 4.2. pH-Responsive

pH-responsive hydrogels control drug release by undergoing expansion, contraction, or degradation in response to environmental pH changes, facilitated by pH-sensitive bonds. For instance, Schiff bases and borate ester bonds hydrolyze in mildly acidic conditions (e.g., pH 5.0), promoting drug release [71,72]. Acylhydrazone bonds can also trigger structural changes in both acidic and alkaline settings, affecting drug diffusion [73]. Like temperature-responsive hydrogels, these hydrogels’ network structures expand or contract with pH variations, in alkaline conditions, expansion increases, pore size enlarges, and drug diffusion is enhanced [74], in acidic environments (e.g., pH 3.0 or 5.4), release speeds up to meet microenvironmental needs [75]. Under acidic conditions, drug release is primarily governed by simple diffusion within the hydrogel matrix, yielding relatively steady release rates. In neutral or alkaline conditions, diffusion and hydrogel relaxation work together. This mechanism is vital in gastrointestinal settings, ensuring rapid release in intestinal fluid (pH 7.4) while protecting drugs in gastric juice (pH 1.2) [76]. Release kinetics studies show marked pH dependence, at pH 1.2, cumulative release may be as low as 28%, but at pH 7.4, it can reach 97%, at pH 5.4, release may be 2.33 times that in neutral conditions [77,78], enabling pH-responsive hydrogels to meet drug release demands in stomach and intestine environments with varying acidity.

### 4.3. ROS-Responsive

ROS-responsive hydrogels are designed to release therapeutic drugs through the cleavage of ROS-sensitive chemical bonds in the polymer structure. For instance, thioether-based bonds undergo oxidative scission in the presence of reactive oxygen species (ROS) that are overexpressed at pathological sites such as inflamed or injured tissues. This mechanism allows for highly localized and on-demand drug release, minimizing off-target effects [79]. Additionally, nanocarriers functionalized with thioether-containing materials can generate ROS under external stimuli like ultrasound irradiation. The resulting oxidation process disrupts the integrity of the nanocarrier’s network, leading to rapid structural disintegration and subsequently accelerating the release of encapsulated drugs [80].

Although ROS-responsive systems exhibit remarkable sensitivity and specificity for targeted therapy, their performance is closely tied to the concentration of ROS at the target site. Key factors such as the threshold levels of hydrogen peroxide (H_2_O_2_) must be carefully evaluated to ensure efficient activation. To enhance responsiveness even under conditions of low ROS abundance, hydrogels can be engineered to incorporate catalytic enzymes such as recombinant unspecific peroxygenase (rUPO). These enzymes significantly accelerate the oxidation of thioether groups, enabling the hydrogel to trigger drug release effectively even at reduced H_2_O_2_ concentrations [81]. Through controlled collapse of the hydrogel matrix, this approach allows fine-tuning of release kinetics and improves therapeutic outcomes. This mechanism underscores the biodegradability and environmental responsiveness of hydrogels, making them ideal for treating chronic diseases.

### 4.4. Light-Responsive Type

Photolysis is a prevalent release mechanism in light-responsive hydrogels, where specific light wavelengths, like near-infrared (NIR) or ultraviolet (UV), cleave photosensitive chemical bonds within the hydrogel [82]. In NIR-responsive hydrogels, coumarin ester groups break under NIR irradiation, causing the hydrogel network to dissociate and release drugs such as doxorubicin (DOX) [83]. This mechanism is extensively used in antitumor therapy, enabling precise spatiotemporal drug release [84]. Similarly, photolyzable linkers can bind drugs like epidermal growth factor (EGF), upon light exposure, these linkers break, allowing on-demand drug release with high control efficiency in wound healing [85]. Photoinduction can also trigger molecular conformational changes, such as the photoisomerization of azobenzene compounds. Light-responsive hydrogels based on this mechanism adjust their porosity or hydrophilicity/hydrophobicity when exposed to light, enhancing network permeability and drug diffusion, making them ideal for targeted cancer therapy delivery [86].

### 4.5. Enzyme-Responsive Type

Enzyme-responsive hydrogels are designed to release therapeutic agents through a mechanism involving enzymatic hydrolysis of internal chemical bonds, which leads to the breakdown or collapse of the cross-linked network structure. A representative example is the action of β-lactamase, which specifically cleaves β-lactam cross-linking agents embedded within the hydrogel matrix. This enzymatic cleavage results in significant changes to the hydrogel’s physical properties, such as its mass and structural integrity, facilitating the controlled release of encapsulated nanoparticles [87]. The responsiveness of such systems is highly specific, as the degradation process is initiated only in the presence of target enzymes, thereby improving the precision and localization of drug delivery. Furthermore, enzymatic reactions can exert indirect control over the hydrogel’s behavior by modulating microenvironmental conditions—such as pH or ionic strength—which in turn influence the swelling or shrinkage of the polymer network. This modulation allows for fine-tuned regulation of drug release kinetics. By integrating the rapid response characteristics typical of physically cross-linked hydrogels with the high biocompatibility of enzyme-mediated processes, enzyme-responsive hydrogels represent a promising platform for achieving targeted and efficient drug delivery in biomedical applications [88].

### 4.6. Double or Multiple Responses

To better align with the intricate dynamics of disease progression, researchers have created smart systems capable of responding to multiple stimuli simultaneously. For instance, pH-responsive hydrogels can be activated by pH shifts caused by glucose oxidase-catalyzed reactions, altering stiffness or relaxing the network for on-demand drug release [89]. Photocrosslinked hydrogels, made from methyl acrylate dextran and polylactic acid, can be triggered by light to activate photosensitizers, generating ROS that induce hydrogel degradation or crosslink disruption, releasing encapsulated drugs [90]. Alternatively, combining thermoresponsive polymers like methylcellulose with ion-responsive materials such as alginate can form a core–shell structure via orthogonal gelation. Elevated temperatures cause hydrophobic aggregation of methylcellulose, while ion crosslinking reinforces the alginate network, enabling controlled release of drug nanocrystals [91]. Near-infrared light irradiation heats MXene and MoO_2_ nanoparticles, causing thermal-responsive polymers to contract, and pH-sensitive borate esters to dissociate in acidic environments, accelerating drug release through dual stimulation [92,93,94]. These mechanisms, achieved through material chemistry design, provide a foundation for smart drug delivery tailored to disease microenvironments.

The pathobiological characteristics of rheumatoid arthritis (RA) (inflammation, acidity, oxidative stress, and abnormal enzyme activity) exhibit a “signal-response” mechanistic relationship with the stimulatory response behavior of smart hydrogels. Through reverse engineering, material scientists of hydrogels transform biochemical markers of disease into physicochemical switches for controlled drug release. The essence of this design logic lies in endowing drug delivery systems with the capability to “perceive” disease states and “execute” therapeutic commands, thereby transitioning from traditional passive systemic administration to active, localized, and on-demand precision therapy [95]. This represents the core innovation and advantage of smart hydrogels in the treatment of RA.

In the treatment of rheumatoid arthritis, stimulus-responsive drug delivery systems can precisely identify the characteristics of the joint inflammatory microenvironment. The synovial fluid in RA joints is acidic, and the pH-responsive system can trigger drug release in this microenvironment. Moreover, the level of reactive oxygen species (ROS) in RA joints is significantly elevated, and redox-sensitive bonds (such as disulfide and trisulfide bonds) can break under these conditions to release drugs [96]. These two mechanisms can work together in the response system, as exemplified by systems constructed with hyaluronic acid cross-linked with alginate and chitosan, loaded with methotrexate (MTX). Under the dual stimulation of the acidic environment and high ROS levels in RA joints, these systems enable precise drug release, promote the polarization of macrophages toward an anti-inflammatory M2 phenotype, downregulate inflammatory factors such as TNF-α, and exhibit both morphological adaptability and self-repair capabilities [97]. RA synovium exhibits high expression of specific enzymes (such as matrix metalloproteinases and proteases), and the enzyme-responsive system enables site-specific drug release. Based on this characteristic, the CIA rat model demonstrated significant reduction in joint inflammation and damage through modulation of the NR4A1 and TET2 pathways, with therapeutic efficacy superior to existing treatments [98]. Thermosensitive hydrogels achieve in situ gelation and controlled release by utilizing body temperature or local thermosensitivity. For instance, liposomes loaded with dexamethasone and safflower I, modified with dextran sulfate, were targeted to macrophages, significantly reducing inflammatory factors, alleviating erythema and bone erosion in rat models, demonstrating temperature-responsive and synergistic therapeutic advantages [99]. In summary, the stimulus-response mechanism provides a novel strategy for RA treatment through the logical chain of “identifying the lesion microenvironment—triggering precise drug release—modulating pathological progression,” offering minimally invasive, long-lasting, and targeted approaches.

## 5. Application of Smart Hydrogels in Rheumatoid Arthritis

With the continuous advancement of gel technology, it has demonstrated significant potential in medical research as a novel strategy for arthritis treatment. The injectability, self-healing properties, and biocompatibility of hydrogels make them suitable for intra-articular applications. Experimental evidence has shown that composite hydrogels (e.g., those incorporating nanomaterials) can simultaneously deliver multiple drugs (such as DMARDs or immunomodulators), thereby enhancing therapeutic efficacy [100].

In the inflammatory joint environment of rheumatoid arthritis (RA), smart hydrogels achieve precise and localized drug release through four core molecular and physicochemical mechanisms. Diffusion control enables passive diffusion and release of drug molecules via the three-dimensional network pores of the hydrogel, driven by concentration gradients. This process does not rely on structural changes in the material and represents a physicochemical-controlled release. The release behavior is stable, with the rate determined by the compatibility between the drug molecule size and the hydrogel network pore size. While this mechanism is easy to simulate in vitro, it struggles to respond to dynamic inflammatory changes and is prone to initial burst release. Swelling control depends on the hydrophilicity of the polymer chains and the crosslinking density, representing a physicochemical-responsive process. When hydrogel networks swell due to environmental stimuli (e.g., pH or ionic strength changes), the expanded pores accelerate drug release. For instance, some hydrogels exhibit enhanced swelling at physiological pH (7.4), resulting in faster release rates compared to acidic conditions, while others demonstrate more pronounced swelling in acidic environments. The swelling-release kinetics of certain materials are complex, often leading to uneven drug release [101]. Degradation-mediated release refers to the gradual disintegration of hydrogel scaffolds through hydrolysis, enzymatic degradation (e.g., MMP-2/9-specific cleavage of peptide bonds), or oxidative degradation, with drug release occurring as the network collapses. The release rate is closely coupled with the degradation kinetics of the material. For instance, MMP-sensitive hydrogels accelerate degradation in inflamed joints due to elevated enzyme concentrations [102]. This process involves chemical bond cleavage, thus belonging to a chemically or enzymatically dominated mechanism. The degradation rate of this mechanism is significantly influenced by individual enzyme activity and metabolic variations; non-specific degradation may lead to premature drug release. Stimulus-induced release utilizes the unique microenvironmental signals of RA joints (such as acidic pH, high reactive oxygen species (ROS), MMP overexpression, and redox imbalance) to trigger structural changes. This mechanism relies on molecular recognition and conformational alterations to achieve “lesion-activated” drug release. Therefore, the concentration of specific stimuli and the complexity of the microenvironment (coexistence of multiple factors) may interfere with the triggering efficiency. Additionally, this mechanism is highly complex in design, requiring solutions to the challenge of microenvironmental heterogeneity on predictability. Through the synergistic interaction of these mechanisms, the limitations of single-release mechanisms in smart hydrogels are reduced, significantly enhancing the targeting and safety of RA localized therapy. Currently, smart hydrogels are widely applied in the treatment of RA. The following lists various applications of responsive hydrogels in RA (Table 1).

### 5.1. Temperature-Responsive Hydrogels for the Treatment of RA

The gel exhibits distinct states at different temperatures influenced by the preparation materials, enabling temperature-controlled drug storage or release. For instance, a hydrogel prepared from chitosan-glycerin-borax becomes a flowable liquid at room temperature. When injected into the joint cavity, it rapidly transforms into a semi-solid gel at body temperature (37 °C). Experimental results demonstrated that the treatment group receiving this gel injection showed reduced levels of PGE2 and PGI2, while PGD2 levels increased. Compared to the control group without gel, the expression of NF-κB p65, IκBα, IKKα, and IKKβ proteins was significantly downregulated [103], confirming the feasibility of anti-inflammatory drug-loaded hydrogels for therapeutic applications. The temperature-responsive mechanism of this hydrogel enables two-phase drug release: rapid partial release within 24 h followed by sustained release of approximately 50% of the drug over 10 days, effectively addressing acute flare-ups in rheumatoid arthritis (RA) and subsequent chronic therapy. This approach significantly downregulates the expression of inflammatory genes such as COX-2, TNF-α, and 18S rRNA [104]. RNA-based drugs and small-molecule inhibitors play a critical role in modulating the immune microenvironment. For example, a hydrogel composed of chondroitin sulfate, chitosan, and sodium β-glycerophosphate, which encapsulates siRNA, exhibits a flowable liquid state at room temperature with low viscosity, allowing minimally invasive injection into the joint cavity. The gelation temperature is 32.06 ± 0.37 °C, aligning with human physiological conditions. The physiological temperature enables rapid transition from liquid to gel within 1 min post-injection, forming a “local drug reservoir.” Subsequent in vivo imaging demonstrated sustained fluorescence signals for >7 days (compared to the disappearance within 3 days in the Fn/siHMGB1 group alone). This prevents the rapid release and degradation observed with RNA drugs used alone, thereby enhancing the bioavailability of gene therapies [105]. The hybrid system composed of aldehyde-terminated polyether F127 and poly-L-glutamic acid ADH (γ-PGA-ADH) exists as a flowing solution at 4 °C, facilitating the loading of cryopreserved drugs. Upon injection into the joint cavity, the Schiff base cross-linking reaction occurs rapidly at body temperature, forming a semi-solid gel within 50–70 s to immobilize the drug locally in the joint, preventing rapid diffusion and loss. The liposomes are continuously released through the Fick diffusion mechanism, and the drug encapsulated within the liposomes remains protected from enzymatic degradation during targeted delivery, further enhancing its stability [106].

### 5.2. pH-Responsive Hydrogels for the Treatment of RA

In the study of hydrogel injection delivery, an experimental method combining a hydrogel composed of chitosan (CS), sodium β-glycerophosphate (β-GP), and hyaluronic acid (HA) with a hollow honeycomb acupuncture needle was employed. Under neutral conditions (pH 7.4), the hydrogel structure remained stable, and drug release was slow. In acidic conditions (pH 5.5, simulating the microenvironment of rheumatoid arthritis [RA] joints), the carboxyl ionization of HA was enhanced, leading to increased negative charge density and subsequent disintegration of the hydrogel network, thereby releasing the drug. Comparative experiments demonstrated that acupoint injection (ST36) maintained local drug concentration more persistently than non-acupoint injection. The binding affinity of MLT to targets such as AKT, JAK2, and SYK was low (<−5 kcal/mol) and stable, which increased the proportion of Treg and M2 macrophages [107]. This method improved the drug-loading efficiency of pH-responsive hydrogels and provided an innovative technical platform for integrated traditional Chinese and Western medicine in treating RA. To test the sustained-release capability of pH-responsive hydrogels, researchers used siMP (siRNA/MTX-PEI complex) and BiMP (bismuth nanosheets/MTX-PEI complex) encapsulated in Nap-GFFKGRH (Nap peptide) for experiments. The results showed that within 7 days, the cumulative release rates of siMP and BiMP at pH 6.5 reached 93.57% and 83.47%, respectively, demonstrating excellent pH responsiveness and complete release potential [108].

Hydrogels offer multiple drug delivery methods, including transdermal administration in addition to injectable routes. For instance, a pH-responsive hydrogel formulated with carbomer can be absorbed subcutaneously to reach the therapeutic site. Under normal human skin and blood conditions, the pH of 7.4 maintains the stability of the liposome structure, releasing only approximately 20% of the drug within 48 h, thereby reducing systemic diffusion and adverse effects. Upon reaching the inflammatory microenvironment with a pH of 6.5, the cationic lipid exhibits increased solubility under acidic conditions, leading to a decrease in liposome membrane stability. This results in an initial burst release of 33% of the drug (within 5 h) followed by sustained release up to 90% cumulatively over 48 h, achieving targeted drug release at the inflammatory site. Transdermal delivery is a non-invasive approach that avoids the first-pass effect in the liver and gastrointestinal irritation. Additionally, carbomer hydrogels can prolong drug retention in the skin (approximately 6.8 h), enabling sustained release [109]. The acidic environment of the gastrointestinal tract is well-suited to the release mechanism of pH-responsive hydrogels, which modulate drug release in response to pH changes to achieve therapeutic goals. For example, S-G hydrogels self-assembled from two solutions containing 100 mM vinpavir and 50 mM glycyrrhizic acid at 80 °C remain structurally stable in the acidic gastric environment (pH 2.0), protecting vinpavir from premature release and degradation. Upon entering the intestinal environment (pH 7.0), the hydrogel gradually dissociates, enabling sustained drug release. Tetrandrine and glycyrrhizic acid. Tetrandrine itself has a short half-life, and the S-G hydrogel prolongs its duration of action in vivo through sustained-release effects, thereby enhancing bioavailability [110].

Nanoparticles, like hydrogels, represent an emerging strategy in modern medical therapy, and their synergistic effects demonstrate significant potential, namely “nanomedicine-hydrogel complexes.” For instance, drug-loaded cationic nanoparticles (cRNPs) can be encapsulated within PEG-based hydrogels. This polymer self-assembles into multibubble micelles at pH 7.4, forming cRNPs that exhibit both binding affinity for cfDNA and pH responsiveness. Under acidic conditions (pH 5.8, such as in endosomes or inflammatory tissues), the PDPA chains in cRNPs become protonated, leading to a reduction in cRNPs size while the hydrogel network slightly swells, thereby accelerating drug release (76% release within 24 h) and achieving targeted drug delivery at the site of interest [111].

### 5.3. ROS-Responsive Hydrogels for the Treatment of RA

Methotrexate (MTX) is a commonly used drug for the treatment of rheumatoid arthritis. However, due to factors such as drug selectivity and bioavailability, long-term administration is required. Hydrogels made from mesoporous polydopamine nanoparticles (MPDANPs) serve as carriers for MTX, enabling sustained release for over 30 days. Moreover, MPDANPs possess the ability to synergistically eliminate reactive oxygen species (ROS). In inflammatory environments with high ROS concentrations, ROS can oxidize the hydrogel network, accelerating MTX release to achieve pathologically responsive drug delivery [112]. ROS are products formed by the combination of oxygen and electrons, including superoxide (O_2_^−^) generated through single-electron reduction, hydrogen peroxide (H_2_O_2_) produced via enzymatic catalysis of superoxide, hydroxyl radicals (·OH) directly generated from the cleavage of hydrogen peroxide catalyzed by transition metal ions (e.g., Fe^2+^, Cu^+^), and nitric oxide (NO) specifically catalyzed by nitric oxide synthase. Researchers will screen a specific ROS from this broad category as the response mechanism of hydrogels to enhance the precision of drug release. For example, through azide-alkyne “click chemistry” in situ gelation, a hydrogel responsive only to NO is fabricated. In rheumatoid arthritis (RA) joints, the excess NO concentration correlates with more severe inflammation. The o-phenylenediamine groups in the gel rapidly react with NO to form benzotriazole derivatives and undergo hydrolysis, leading to the disruption of the gel crosslinking network, enlargement of gel pores, and volume swelling. The higher the NO concentration, the greater the swelling ratio. This provides a pathway for drug release; simultaneously, the mechanical strength of the gel decreases (storage modulus G declines with increasing NO concentration), ultimately degrading gradually at the inflammatory site [113]. This method is deeply integrated with “NO clearance anti-inflammation,” addressing the issues of poor drug targeting, short action duration, and significant systemic side effects in traditional RA treatments.

### 5.4. Light-Responsive Hydrogels for the Treatment of RA

Phototherapy, as a unique physical response mechanism, is often accompanied by thermal reactions. For instance, hydrogels composed of black phosphorus nanosheets (BPNs) can efficiently convert light energy into thermal energy under near-infrared (NIR) irradiation (with a photothermal conversion efficiency of approximately 43.19%), resulting in a local temperature increase of about 25 °C. These hydrogels exhibit excellent cyclic stability (rapid heating/cooling after 4 cycles) and the ROS generated by BPNs under light exposure are cytotoxic, specifically killing abnormal synovial cells while alleviating local inflammatory responses [114]. Hyaluronic acid surface-modified gold nanoparticles (AuNPs) hydrogels show a 25% increase in drug release rate from 25% in the absence of light to 55% under NIR irradiation, with thermotherapy reducing local TNF-α levels in joints by 40% [115]. Additionally, NIR irradiation can induce photoelectric effects. For example, composite hydrogels composed of polypyrrole nanoparticles (Ppy NPs) and molybdenum disulfide nanoflakes (MoS_2_ NFs) generate photogenerated carriers under NIR irradiation, producing detectable current changes (verified by ITO substrate testing, with stable current after multiple irradiation cycles). This photoelectric effect can modulate the membrane potential and signaling pathways of immune cells (e.g., macrophages). The photoelectric signals can directly regulate the polarization direction of macrophages, inhibiting the generation of pro-inflammatory M1-type and promoting anti-inflammatory M2-type macrophages, thereby alleviating local immune dysregulation in rheumatoid arthritis (RA). The composite hydrogel composed of alginic acid (ALG) and MoS_2_ nanofibers (NFs) loaded with drugs exhibits minimal drug release (<10%) in the absence of NIR irradiation, as the ALG gel network binds the drugs through hydrogen bonding and electrostatic interactions. After NIR irradiation, the photothermal effect causes slight contraction of the ALG gel network, while the photoelectric effect of MoS_2_ NFs promotes gel degradation. Together, these two mechanisms trigger “on-demand release” of the drug [116].

### 5.5. Enzyme-Responsive Hydrogels for the Treatment of RA

HA is a natural component of synovial fluid, exhibiting lubricating, biocompatible, and cartilage-protective properties. Due to the presence of hyaluronidase in the joint cavity, hydrogels based on HA can utilize enzymatic reactions to control drug release. For instance, the combination of HA and TLR4 antagonist peptide (CP) produces different enzymatic response mechanisms depending on the preparation method: in physically mixed hydrogels, HA and CP are not chemically bonded. After HA is degraded by hyaluronidase, CP rapidly diffuses and releases, resulting in a shorter retention time. In chemically coupled hydrogels, CP is bonded to HA through chemical bonds, slowing the degradation rate of HA by hyaluronidase and further hindering CP diffusion via the gel network, significantly prolonging CP retention in the joint (up to 42 days in vivo, which is over 19 times longer than CP administered alone). This formulation targets the pathological mechanisms of rheumatoid arthritis (RA) by achieving “local sustained-release of TLR4 antagonists and anti-inflammatory repair” through the binding of CP to TLR4, thereby blocking its downstream signaling pathways [117]. To regulate the release of interleukin-4 (IL-4), researchers selected HA as the substrate and prepared it through a three-step process: “precursor synthesis → IL-4 conjugation → cross-linking into a gel.” The core optimization focused on mechanical properties and retention of biological activity. Specifically, the HA-VS hybrid formulation is fluid under normal conditions but, when mixed with polyethylene glycol dithiol (PEG-dithiol) in a specific ratio, the vinylsulfone groups of HA-VS interact with PEG-dithiol to form a stable structure. The thiol groups undergo rapid Michael addition reactions, forming a three-dimensional network structure that transforms the sol into a gel. The gelation process takes approximately 2 min, during which the material can be injected into the joint cavity. Hyaluronidase in the joint cavity gradually degrades HA, progressively releasing covalently coupled IL-4. Compared to traditional physical encapsulation methods, this approach avoids the sudden release of free IL-4, enabling its long-term stable presence in the joint and extending the therapeutic window [118].

### 5.6. Dual or Multiple Response Hydrogels for RA Treatment

Certain hydrogels exhibit multiple response mechanisms due to their unique material properties or synthesis methods, which are interconnected and collectively function during the RA process. For instance, hydrogels targeting phagocytic synovial cells can utilize N-(2-hydroxypropyl)methylacrylamide (HPMA) copolymers as temperature-responsive materials. These materials form a gel in the joint cavity and release the drug slowly. The released prodrug is preferentially phagocytosed by cells, followed by the cleavage of the hydrazone bond triggered by intracellular lysosomes, which releases the free active drug to inhibit the secretion of pro-inflammatory factors by synovial cells, thereby reducing inflammatory cell infiltration, alleviating joint swelling, and mitigating synovitis [119].

Hydrogels can also be combined with other responsive materials to achieve sustained drug release. The HRV@gel, composed of a “HA-based hydrogel carrier” and “HRV nanovaccine,” exhibits “enzyme-responsive degradation at the hydrogel level” and “intracellular dual-response release at the HRV level,” ensuring precise and sustained drug delivery. First, hyaluronidase within the joint cavity disrupts the three-dimensional porous structure of the hydrogel, allowing the encapsulated HRV to be slowly released. Upon entering dendritic cells (DCs), the HRV releases its active components through two mechanisms: intracellular high concentrations of glutathione (GSH) cleave the disulfide bonds on the HRV membrane, leading to vesicle de-crosslinking and release of rapamycin; tissue proteinase B (lysosomal) or protease K (cytoplasmic) within DCs cleave the GPGPG linker between the heat shock protein (HSP) peptide and copolymer, releasing free HSP peptide (antigen). The core logic is a therapeutic process of “sustained delivery of nanovaccine → regulation of immune cells → reconstruction of local and peripheral immune tolerance → alleviation of inflammation and joint protection” [120]. The HA-CPT hydrogel, composed of an “HA-based hydrogel carrier” and “camptothecin (CPT) nanocrystal drug,” combines the slow-release properties of both the hydrogel and the drug itself. When the drug is encapsulated by the hydrogel, reactive oxygen species (ROS) at the RA site and transition metal-mediated oxidative degradation are simultaneously affected by the permeability of the hydrogel. The enzymatic degradation by hyaluronidase. As the hydrogel degrades, CPT is gradually exposed to synovial fluid, releasing free CPT through surface dissolution. The dissolution rate is independent of crystal size, and this process proceeds stably for over 4 weeks, with 35% of the hydrogel remaining after this period [121]. This strategy of maintaining local drug concentrations at therapeutic levels for extended periods undoubtedly provides new insights for the research of chronic diseases.

## 6. Future Development Direction and Current Challenges of Smart Hydrogels in RA

### 6.1. Development Direction

Smart hydrogels have demonstrated multifaceted advantages and innovative potential in the treatment of RA. Drug delivery systems are emerging as a pivotal innovation in the biopharmaceutical field, driving the annual expansion of the global medical hydrogel market. The demand for chronic disease management due to population aging, coupled with the integration of intelligent responsive materials (such as temperature-sensitive, pH-sensitive, and enzyme-sensitive hydrogels) and bioprinting technologies, is propelling the development of personalized and adaptive therapies. The industry trends and investment interests primarily revolve around precision, intelligence, and long-term efficacy [122].

Future research will continue to develop smart hydrogels capable of real-time sensing and responding to pathological microenvironmental changes within the joint cavity. The research focus includes the following three aspects:

(1) Develop dual-responsive systems by integrating dynamic chemical bond design and advanced nanocomposites, with a focus on achieving precise stimulus-response mechanisms. These systems should be engineered to exhibit co-responses to ROS and pH variations, enabling highly controlled and targeted drug release [123,124]. Additionally, the design should incorporate cascade reactions specifically tailored for inflammatory environments, ensuring on-demand therapeutic delivery in response to pathological conditions [125].

(2) Develop an advanced high-throughput screening system specifically designed for smart hydrogel materials, incorporating integrated microfluidic platforms to establish a dynamic perfusion environment that accurately simulates the physiological conditions of the joint cavity microcirculation [126]. This system allows for precise regulation of inflammatory factor concentration gradients and application of controlled mechanical stress, closely replicating in vivo scenarios. By facilitating efficient evaluation of drug impacts on critical pathological mechanisms—such as macrophage polarization states and synovial fibroblast activation patterns—the platform significantly accelerates the development and optimization of hydrogel-based therapies for targeted applications [127]. This approach not only enhances screening efficiency but also promotes the adoption of industrial-scale microfluidic technologies in biomedical research and production.

(3) Develop a “Therapeutic-Repair-Regenerative” smart Hydrogel System, Given RA’s heterogeneity, integrating genomic, proteomic, and metabolomic data is crucial for analyzing patient subgroups [128]. This transforms hydrogels from simple drug carriers to multifunctional platforms with therapeutic, repair, and regenerative capabilities. For example, photothermal-responsive hydrogels can release antibiotics under near-infrared light to fight infections, while carrying antioxidants like curcumin to reduce inflammation by scavenging reactive oxygen species [129]. This multifunctional system, combining photothermal antibacterial, antioxidant, and angiogenic properties, may address challenges like degradation synchronicity and clinical applicability, enabling precise tissue regeneration.

### 6.2. Key Technologies Related to Hydrogel and Rheumatoid Arthritis (RA) Stimulus-Response Delivery Platforms

(1) Dual-sensitivity hyaluronic acid-chondroitin sulfate nanoplatform: This technology developed a pH/ROS dual-responsive nanodelivery system for the synergistic intra-articular delivery of glucosamine (for cartilage repair) and tofacitinib (for anti-inflammatory effects) in rheumatoid arthritis (RA). The system triggers glucosamine release in the acidic joint microenvironment, where macrophages recognize and engulf the glucosamine via CS-CD44. Intracellular reactive oxygen species (ROS) then induce nanoparticle disintegration and rapid drug release, promoting M1-to-M2 macrophage polarization to alleviate inflammation. Both in vitro and in vivo experiments validated its synergistic therapeutic effects, representing a pivotal patented technology in responsive delivery platforms [130].

(2) Acupoint Nanocomposite Hydrogel Delivery System: The nanocomposite hydrogel constructed based on acupoint drug delivery strategy can target the delivery of Tripterygium wilfordii saponin and CCPA to RA lesions. Studies have demonstrated that it significantly enhances drug accumulation in joint tissues (a 13.5-fold increase within 48 h), improves analgesic effects (with mechanical and thermal pain thresholds increased by 4.9-fold and 1.6-fold, respectively), alleviates inflammation and bone erosion, while reducing systemic toxicity. This study provides important technical references for hydrogel composition and rheumatoid arthritis stimulus-responsive delivery platforms [131].

### 6.3. Current Challenges

**Figure 4:**
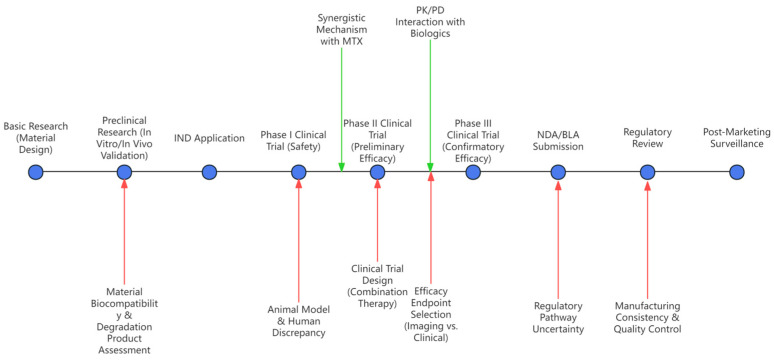
A new drug development flowchart combining MTX with smart hydrogel as an example.

Although smart hydrogels demonstrate significant potential in RA treatment due to their unique drug delivery systems, their clinical translation still faces multiple challenges. There is no unified standard for long-term biocompatibility and safety evaluation of hydrogel materials. Existing studies predominantly focus on short-term efficacy, while the cumulative toxicity of material degradation products, local inflammatory responses, and potential immunogenicity lack systematic tracking [132]. For instance, the immune response triggered by polyethylene glycol (PEG) and long-term biosafety concerns arising from chemical cross-linking agent residues; their degradation kinetics are influenced by interactions between the material and the physiological environment, exhibiting unpredictability [133]. The use of amphoteric materials or natural polymer-based hydrogels can reduce the immunogenicity and the residues of chemical crosslinking agents. However, systematic long-term safety assessments (e.g., chronic toxicity, immunogenicity, material degradation kinetics) and standardized model development (e.g., unified animal models, evaluation metrics, follow-up cycles) remain research gaps. Additionally, some hydrogels rely on synthetic polymer materials (e.g., polyaminopropionic acid), and the long-term effects of their residual metabolites on joint tissues require further clarification [134,135]. The complex composition and structural design of hydrogels also pose technical bottlenecks for large-scale production. Functional hydrogels (e.g., drug/gene co-delivery nanocomposites) require precise control of crosslinking density, drug-loading uniformity, and batch stability, which current microfluidic technologies still struggle to meet industrial production demands. Meanwhile, standardized processes for sterile filling and low-temperature storage remain incomplete, hindering clinical translation [136]. As illustrated in the Figure 4, the journey from R&D to market launch remains a lengthy process.

In summary, overcoming these translation bottlenecks requires multidisciplinary collaboration to establish standardized evaluation systems, optimize production processes, and integrate multi-omics data to guide personalized application scenarios.

## Figures and Tables

**Table 1:** Application of Smart Hydrogels in RA.

Types of Smart Hydrogels	Drug Loading	Therapeutic Principles	References
temperature responsive	Dexamethasone	ProGel-Dex forms a hydrogel at 30 °C, which is recognized by synovial cells and endocytosed. Under the acidic environment of lysosomes, the hydrazone bond is cleaved, releasing free dexamethasone to exert local anti-inflammatory effects.	[103]
	Diclofenac Sodium	Downregulation of key inflammatory genes such as TNF-α, COX-2, and 18S rRNA, inhibition of pathways including NF-κB, and reduction in pro-inflammatory cytokine release. Hydrogels form a lubricating layer within the joint, reducing interosseous friction and alleviating pain.	[104]
	siHMGB1	Silencing HMGB1 gene expression regulates macrophage polarization and inhibits inflammatory pathways.	[105]
	SNH (Hydrochloride of Coptisine)	Inhibition of macrophage polarization and reversal of the inflammatory microenvironment.	[106]
pH-responsive	Melittin (MLT)	Regulation of immune cells: Promotes the proliferation of M2-type macrophages (anti-inflammatory) and inhibits M1-type macrophages (pro-inflammatory), while suppressing the NF-κB and AKT signaling pathways.	[107]
	MTX-PEI, siRNA (siCD86, sip65, sip38), BiNS	Inhibition of dendritic cell antigen presentation and clearance of hyperproliferated FLS.	[108]
	Dexamethasone	Inhibit the secretion of pro-inflammatory factors and reduce synovial hyperplasia.	[109]
	Veratrine (SIN), Glycyrrhizic Acid (GA)	Inhibit inflammatory signaling pathways and reduce the production of inflammatory factors.	[110]
	cGAS inhibitor, cfDNA scavenger	Inhibition of cGAS, clearance of cfDNA, and regulation of T cell subsets.	[111]
ROS responsive	Methotrexate (MTX)	Inhibition of ROS-mediated NF-κB signaling pathway, reduction in pro-inflammatory factor expression, promotion of macrophage polarization from pro-inflammatory M1 to anti-inflammatory M2, and alleviation of joint swelling, synovial hyperplasia, and cartilage destruction.	[112]
	Dexamethasone	Clear excess NO, synergize with drugs to reduce pro-inflammatory cytokines.	[113]
photoresponsive	Methotrexate	Promote bone regeneration, remove hyperplastic synovial tissue, and eliminate abnormal cells.	[114]
	Triptolide	Enhance drug concentration at the lesion site to inhibit the pathological progression of RA.	[115]
	Strontium Ranelate	MoS_2_ nanofibers generate photoelectric signals under NIR irradiation, triggering the on-demand release of strontium reneine to modulate the function of local immune cells (e.g., macrophages) and promote their polarization toward an anti-inflammatory phenotype (M2 type).	[116]
enzyme-responsive	TLR4 antagonist peptide	Block inflammatory signaling pathways and repair damaged joint tissues.	[117]
	Interleukin-4 (IL-4)	Activates the STAT6 signaling pathway, promotes macrophage polarization, and enhances local immune regulatory effects.	[118]
dual or multiple response	Dexamethasone	Inhibition of pro-inflammatory factors and release of small molecules associated with pain pathways.	[119]
	Rapamycin, heat shock protein peptide	Regulate immune cells to inhibit articular cartilage damage and bone erosion.	[120]
	camptothecin	Inhibits synovial cell DNA replication, suppresses their proliferation and invasion, and simultaneously inhibits angiogenesis to alleviate joint inflammation.	[121]

## Data Availability

No new data were created or analyzed in this study.

## Abbreviations

The following abbreviations are used in this manuscript:

RA	Rheumatoid Arthritis
ILD	Interstitial Lung Disease
DMARDs	Disease-modifying Antirheumatic Drugs
NSAIDs	Nonsteroidal Anti-inflammatory Drugs
ICIs	Inflammatory Cytokine Inhibitors
TNF-α	Tumour Necrosis Factor-alpha
IL-6	Interleukin-6
ROR	Rapid Onset Rate
NF-κB	Nuclear Factor Kappa-B
FLS	Fibroblast-like Synovial Cells
MMPs	Matrix Metalloproteinases
IL-23	Interleukin-23
IL-17	Interleukin-17
Th17	T helper cell 17
DNA	DeoxyriboNucleic Acid
cGAS	Cyclic GMP-AMP synthase
AGE-RAGE	Advanced glycation end product and receptor for Advanced Glycation End Products
HIF-1	Hypoxia-inducible factor-1
SASP	Senescence-associated secretory phenotype
CCL20	C-C motif chemokine ligand 20
VEGF	Vascular endothelial growth factor
JAK-STAT	Janus kinase-signal transducer and activator of transcription
BMP	Bone Morphogenetic Protein
RAM	Rheumatoid Arthritis Microenvironment
PVA	Polyvinyl Alcohol
HA	Hyaluronic Acid
DHPA	Dihydroxyphenylacetic Acid
NFs	Nanofibers
CNFs	Carbon Nanofibers
NIPAAm	N-Isopropyl-2-aminoacetamide
ELP	Elastin-like Polypeptides
LCST	Low Critical Solubility Transition
ROS	Reactive Oxygen Species
rUPO	recombinant Unspecific Peroxygenase
NIR	Near-infrared
UV	Ultraviolet
DOX	Doxorubicin
EGF	Epidermal Growth Factor
MXene	Methoxyisopropylisopropyl
CIA	Collagen-Induced Arthritis
NR4A1	Nuclear Receptor Subfamily 4 Group A Member 1
TET2	Ten-Eleven Translocation 2
PGE2	Prostaglandin E2
PGI2	Epoprostenol
PGD2	Prostaglandin D2
NF-κB p65	Nuclear factor kappa-light-chain-enhancer of activated B cells p65 subunit
IκBα	Nuclear factor of kappa light polypeptide gene enhancer in B-cells inhibitor, alpha
IKK	Inhibitor of Kappa B Kinase
COX-2	Cyclooxygenase-2
RNA	Ribonucleic Acid
siRNA	Small interfering RNA
Fn	Fibrin
siHMGB1	Small interfering High Mobility Group Box 1 Protein
PGA	poly-L-glutamic acid
ADH	Alcohol DeHydrogenase
Ppy NPs	Chitosan
β-GP	β-glycerophosphate
MLT	Melittin
AKT	Protein Kinase
SYK	Spleen Tyrosine Kinase
MTX	Methotrexate
PEI	Polyethyleneimine
cRNPs	Cationic Nanoparticles
MPDANPs	Mesoporous Polydopamine Nanoparticles
BPNs	Black Phosphorus Nanosheets
AuNPs	Gold Nanoparticles
Ppy NPs	Polypyrrole Nanoparticles
ITO	Indium Tin Oxide
ALG	Alginic Acid
TLR4	Toll-like receptor 4
CP	TLR4 antagonist Peptide
PEG	Polyethylene Glycol
HPMA	Hydroxypropyl Methacrylate
HRV	Human Coronavirus
DCs	Dendritic Cells
GSH	Glutathione
HSP	Heat Shock Protein
CPT	Camptothecin
IND	Investigational New Drug
PK	Pharmacokinetics
PD	Pharmacodynamics
NDA	New Drug Product Approval
BLA	Biologics License Application
